# Multidimensional empirical research on fertility motivation and influencing factors of Chinese university students

**DOI:** 10.1016/j.isci.2026.117059

**Published:** 2026-08-07

**Authors:** Zitong Guo, Xinmin Zhang, Jinpei Chen, Wenxuan Liang, Lina Song, Yongai Zhang

**Affiliations:** 1 Medical School, Yan’an University, Yan’an, China; 2 College of Nursing Science, Kyung Hee University, 26 Kyungheedae-ro, Dongdaemun-gu, 02447 Seoul, Republic of Korea; 3 School of Nursing and Rehabilitation, Xi’an Medical University, Xi’an, China; 4 Pomegranate Flower (Xi’an, Shaanxi Province, China) Medical Management Co., Ltd, Xi'an, Shaanxi Province, China

**Keywords:** fertility motivation, college students, structural equation model, influencing factors, fertility knowledge

## Abstract

This study explored factors influencing fertility motivation among Chinese college students and provided empirical evidence for understanding young people’s fertility intentions. From July to August 2025, using a multi-stage cluster sampling method, a questionnaire survey was conducted across 12 universities in 9 provinces (including municipalities and autonomous regions) of China. Among the valid samples, 238 individuals clearly expressed their willingness to have children in the future, accounting for 15.3%. The model explained 29.7% of the variance in fertility motivation. Path analysis indicated that of all the included variables, reproductive knowledge exhibited the highest standardized total association coefficient of 0.331 with fertility motivation, including a direct effect of 0.294 and an indirect effect of 0.037. Based on correlation analysis, this study identifies factors related to college students' fertility motivation, which can help us better understand and support the development of their fertility motivation, while also providing references for potential future intervention directions.

## Highlights

• Chinese college students' fertility motivation is at a moderate level
• There is a certain correlation between fertility knowledge and fertility motivation
• Analyzing college student characteristics to inform future interventions and research

## Graphical abstract

**Figure:**
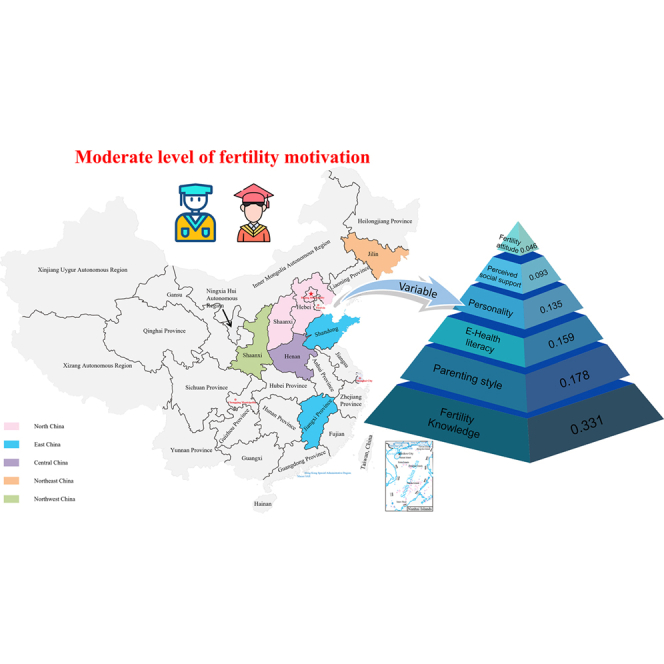

## Teaser

Reproductive medicine; Psychology

## Introduction

China’s current population structure exhibits complex features of negative growth, aging, and high mobility, which has also become a widely - concerned hot topic across all sectors of Chinese society.^1^ The evolving demographic landscape forms the practical foundation for the optimization of population policies. In light of this, China’s population policy has gradually evolved from the early one-child policy to the “three-child” policy implemented in 2021. This series of policy changes also reflects China’s proactive response to population development. The long-term policy environment and fertility restrictions have shaped the fertility concepts and attitudes of contemporary youth; they also exert a certain influence on decisions regarding the number of children, family size, and timing of childbirth. Despite the gradual relaxation of birth restrictions, the actual fertility rate recovers sluggishly, and the tendencies of population negative growth and population aging remain unaltered. The phenomenon of low fertility rates has evolved into a shared challenge confronting numerous countries. Delayed childbearing and low fertility intentions among the youth are widespread social phenomena. To address declining fertility rates, many countries have introduced supportive policies, such as Norway’s parenting support strategy^2^ and Sweden’s paternity leave.^3^ Although China has continuously strengthened fertility support policies, the recovery of the actual fertility level is modest. The low fertility rate is likely to impose significant implications for the long-term balanced population development in China.^4^

Low fertility rates are associated with insufficient reproductive motivation in Homo sapiens. Therefore, exploring pathways to enhance reproductive motivation in Homo sapiens may become one of the research directions for addressing current population issues. Miller^5^ proposed that fertility behavior follows a sequence of “fertility motivation →fertility desire →fertility planning →intermediary behavior →fertility outcome,” thus, fertility motivation serves as the initial link in the fertility decision-making sequence.^6^ Fertility motivation is a core component of fertility concepts, referring to the fundamental reason or initial drive for an individual to have children.^7^ It serves as both the internal driving force and the direct inducement for fertility behavior. Research indicates that more than half of female college students report low fertility intentions.^8^ Notably, as a major participant in childbearing activities in the coming decade, college students’ fertility motivation and concepts are of remarkable social and demographic importance.^9^ Furthermore, their fertility intention and behavioral inclination may considerably affect future population development trends and structural changes.

According to the Statistical Communiqué on Educational Development of the Ministry of Education of the People’s Republic of China, there were 286.465 million students enrolled nationwide in 2024.^10^ Furthermore, China’s gross enrollment rate in higher education exceeded 60% in 2023, forming the largest higher education system globally.^11^ College students are at a pivotal juncture, transitioning from university life to the broader society. Fertility motivation among college students is influenced by multiple related factors, including individual attributes, social environment, and family environment. Compared with married couples and employed youth, college students exhibit a weaker direct perception of practical fertility constraints. Their fertility motivation is more manifested as rational reflection and planning on future childbearing practices, and remains relatively idealized.^12^

The new generation of Chinese youth possesses distinct generational characteristics and is greatly influenced by the concepts of individualism and gender equality concepts.^13^ They are probably more sensitive to external environmental pressures, ranking among the groups with the strongest perception of pressure of all current generations. Fertility issues are also worthy of attention within this demographic group. Theoretically, the decisions of this group regarding reproduction are probably based on their own predictions and rational evaluations of future marital and reproductive scenarios; thus, it presents relatively rational characteristics, but the specific mechanism remains to be empirically tested. Observation view, under the influence of various factors, the reproductive views of the youth group have undergone changes, which are specifically manifested as a decrease in fertility intentions, an extension of the childbearing age, a shortening of the interval between marriage and childbearing, and a shift in gender preferences for childbirth.^14^ The mechanisms underlying these changes may serve as crucial clues for understanding the youth and are also worthy of further research.

Existing research on fertility motivations mainly focuses on married couples, working young adults, and families with two or three children, while studies targeting college students—a potential fertility group—are limited. Even when research involves youth fertility, it often remains at a macroscopic, descriptive level, lacking a comprehensive analysis of internal driving or hindering factors such as fertility motivation. The neglect of the college student group makes it difficult for us to systematically understand the real fertility demands of young childbearing groups. Moreover, discrepancies exist between international theories and the local Chinese context; the research findings may be difficult to fully align with the actual context of China.

Integrating interdisciplinary perspectives from reproductive health, social psychology, and health communication, this study constructs an interdisciplinary analytical framework around the internal relationships among reproductive knowledge, e-Health Literacy, and fertility motivation. To a certain extent, it enriches the empirical evidence in the field of population and family health, and also provides a reference for understanding the fertility decision-making process from an interdisciplinary perspective. Existing studies have shown that the formation of college students’ fertility motivation is usually jointly influenced by cognitive factors and psychosocial factors, and is associated with variables such as fertility attitudes, parental rearing styles, reproductive knowledge, and e-Health Literacy to varying degrees. The significance of this study is mainly reflected in two aspects: On the one hand, through the analysis of Chinese college students’ fertility motivation, it presents the characteristics of contemporary young people’s fertility concepts, which can provide empirical reference from the Chinese context for international research related to low fertility; on the other hand, the research results may provide insights for other countries and regions facing similar low fertility challenges in formulating fertility support policies targeting young groups.

In recent years, scholars have continuously deepened their research on reproductive cognition. Some researchers conducted surveys among college students,^15^ and the results showed that the reproductive attitudes of college students worldwide were relatively low, and their overall cognitive level was at a medium to low level. A study targeting young people in Denmark revealed^16^ that the participants' knowledge of reproduction was generally at a medium level. This group believed that acquiring reproductive knowledge and methods for protecting reproductive ability was of great importance. They hoped to obtain reproductive knowledge from professionals through various channels and forms.

Regarding social support, studies have shown that women with greater social support demonstrate stronger fertility motivation, particularly when they have an individualistic value orientation.^17^ Beyond tangible resources, such as economic conditions and real estate, family-related factors have also attracted the attention of many experts. Shicun Xu et al.^18^ analyzed the impact of family capital on the fertility intentions of college students and found that it significantly promoted their willingness to have children.

The Internet, as a primary medium for social interaction, emotional expression, and information acquisition in contemporary society, plays an increasingly important role in shaping perceptions of fertility. Some scholars^19^ have found through empirical analysis that the more electronic resources the research subjects obtain from the Internet, the fewer reasons they identify for having children. Consequently, fertility motivation weakens, as individuals become more inclined to accept fertility intentions aligned with personal interests while rejecting family-oriented fertility content.

**Figure 1:**
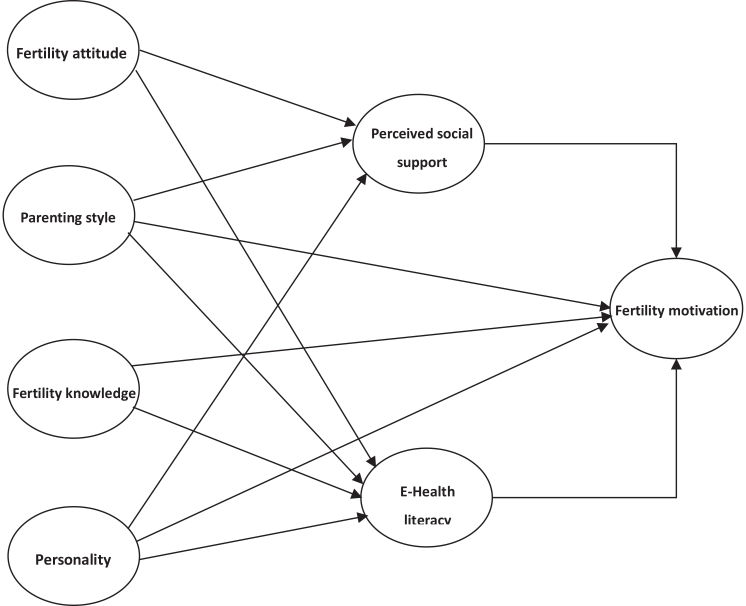
Theoretical framework of structural equation model for college students' fertility motivation This figure illustrates the theoretical framework of this study, where ovals represent latent variables, arrows represent hypothesized causal pathways, and all research hypotheses are formulated based on attribution theory and previous studies.

This study establishes a core framework grounded in Heider’s attribution theory and previous research. By integrating variables such as fertility attitudes, parenting styles, personality, perceived social support, e-Health literacy, and fertility knowledge, a fundamental theoretical framework (Figure 1) was constructed. As a motivational theory, Heider’s theory emphasizes individuals' causal inferences regarding the outcomes of their own behaviors or those of others. At its core, the causes of behavior are divided into two categories: internal causes (dispositional attribution), which encompass subjective elements such as personality, emotions, and fertility attitudes; and external causes (situational attribution), which include objective factors such as the actions and perspectives of others.^20^^,^^21^ Based on this theory, college students’ fertility motivation can be analyzed through the interaction of internal and external factors. From the perspective of internal attribution, personality, e-Health literacy, and fertility attitudes significantly influence the development of fertility motivation. From the perspective of external attribution, parenting styles and social support also play crucial roles.

In addition to attribution theory, existing studies further clarify the path relationships among the variables, as follows.

(1) Fertility Attitude Pathway: In the contemporary online society, individuals exhibit selective behavior in information searching, tending to acquire electronic resources that are consistent with their own fertility attitudes^22^; meanwhile, fertility attitudes are also correlated with perceived social support.^23^
(2) Parenting Style Pathway: As a microcosm of society, the family environment subtly influences offspring’s fertility motivation^18^ as well as their Internet usage and electronic resources-seeking behavior.^24^ Positive parenting styles can also significantly enhance children’s perceived social support, demonstrating a positive correlation between the two.^25^
(3) Fertility Knowledge Pathway: The amount of fertility knowledge directly affects an individual’s fertility motivation;^26^ fertility knowledge among women of childbearing age is also associated with media exposure, such as electronic resources.^19^ At the same time, increased fertility knowledge may intensify fertility-related anxiety, thereby altering fertility motivation.^27^
(4) Personality Pathway: As an important human attribute, personality influences individuals in various ways. A proactive personality, as a positive trait, enables individuals to access more supportive resources from their environment; in other words, personality affects an individual’s perceived social support.^28^ Anxiety-related traits, such as neuroticism and obsessive tendencies, as well as different personality types, are associated with online electronic resources-seeking behavior.^29^ Moreover, personality can significantly influence fertility trends among individuals of reproductive age throughout their life stages. Extroverted personalities tend to facilitate these trends, whereas neurotic personalities may inhibit them.^30^
(5) Mediating Variables: Regarding perceived social support, fertility motivation is positively correlated with the level of support perceived.^17^ In terms of e-Health literacy, the more electronic health resources on fertility an individual obtains from the Internet, the fewer reasons they identify for having children. This ultimately results in weaker fertility motivation.^19^

## Results

### General characteristics

A total of 1,550 valid questionnaires were collected in this study. The participants' average age was 20.39 ± 1.726 years, the participants aged 21 or above accounted for the majority, at 37.3%. Among them, 296 were male (19.1%), and 1,254 were female (80.9%). There were 411 only children (26.5%), while 1,139 were not (73.5%). Regarding household registration, the majority came from rural areas (69.7%). In terms of family size, households with four members were the most common (45.4%). By academic discipline, science majors accounted for 77.7% of the sample, the proportion of liberal arts majors is relatively low, accounting for only 22.3%. As for current fertility intentions, 238 participants (15.3%) expressed a desire to have children in the future, 798 participants (51.5%) were undecided, and 514 participants (33.2%) explicitly stated that they did not want to give birth to a baby in the future. Detailed characteristics are presented in Table 1.

**Table 1:** General characteristics (*n* = 1550)

Factors	Groups	n	Frequency (%)	Mean (SD)
Age	18–19	473	30.5	20.39 ± 1.726
	20	499	32.2	–
	≥21	578	37.3	–
Gender	male	296	19.1	–
	female	1254	80.9	–
Is the individual an only child in your family?	yes	411	26.5	–
	no	1139	73.5	–
Household registration nature	urban household registration	470	30.3	–
	rural household registration	1080	69.7	–
Number of family members	2	38	2.5	–
	3	298	19.2	–
	4	704	45.4	–
	≥4	510	32.9	–
Subject classification	liberal arts	346	22.3	–
	science subjects	1204	77.7	–
Whether to choose to have children in the future?	yes	238	15.3	–
	uncertain	798	51.5	–
	no	514	33.2	–

### Descriptive analysis of fertility motivation

As shown in Table 2, the mean scores for fertility motivation varied significantly: 16.512, 19.906, 16.523, 13.334, and 13.172. Further comparison reveals that the mean score for the utility dimension was substantially higher than that of the other dimensions.

**Table 2:** Status of fertility motivation variable dimensions

Item	Range	Mean
Traditional concepts category	7–25	16.512 ± 4.794
Utility category	7–30	19.906 ± 5.850
Self-esteem category	5–25	16.523 ± 4.930
Harmony category	5–20	13.334 ± 3.896
Love for children category	4–20	13.172 ± 3.924

### Measurement model

The measurement model in this study comprised 7 latent variables and 21 observed variables. The model was validated. All standardized factor loadings exceeded 0.7 (Table 3). Confirmatory factor analysis showed that the average variance extracted (AVE) values for each factor were greater than 0.5, and that the composite reliabilities (C.R.) were all above 0.7,^31^ indicating good convergent validity (Table 3).

**Table 3:** Confirmatory factor analysis

Latent variable	Item	Factor load (FL)	Average variance extracted (AVE)	Construct reliability (CR)
Fertility attitude	X11	0.954	0.935	0.879
	X12	0.923	–	–
Parenting style	X21	0.937	0.962	0.894
	X22	0.936	–	–
	X23	0.930	–	–
Fertility knowledge	X31	0.916	0.923	0.799
	X32	0.908	–	–
	X33	0.883	–	–
Personality	X41	0.721	0.953	0.910
	X42	0.774	–	–
Perceived social support	Y11	0.933	0.931	0.819
	Y12	0.928	–	–
	Y13	0.921	–	–
E-Health literacy	Y21	0.965	0.883	0.717
	Y22	0.837	–	–
	Y23	0.785	–	–

X11, positive; Y11, family support; X12, negative; Y12, friend support; X21, rejection; Y13, other support; X22, emotional warmth; Y21, application ability of test network health information and services; X23, over protection; Y22, judgment ability; X31, indicators of reduced fertility; Y23, decision-making ability; X32, misunderstandings about fertility; Y31, traditional concept category; X33, understanding of infertility; Y32, utility category; X41, introversion and extroversion; Y33, self-esteem category; X42, emotional stability; Y34, harmony category; Y35, love for children category.

According to the Pearson’s correlation coefficients, fertility motivation was positively correlated with fertility attitude, parenting style, fertility knowledge, personality, perceived social support, and e-Health literacy (r = 0.344, *p* < 0.01; r = 0.377, *p* < 0.01; r = 0.450, *p* < 0.01; r = 0.313, *p* < 0.01; r = 0.274, *p* < 0.01; r = 0.367, *p* < 0.01) (Table 4). Discriminant validity was also tested (Table 4). The correlation coefficients among the variables were all less than the square roots of their respective AVE values, confirming good discriminant validity.^32^

**Table 4:** Average scores, discriminant validity and Pearson’s correlation coefficients of each variable item (*n* = 1550, SD, standard deviation)

	Mean (SD)	Range	X1	X2	X3	X4	Y1	Y2	Y3
Fertility attitude (X1)	1.33–5.00	3.36 ± 0.97	**0.937**	–	–	–	–	–	–
Parenting style (X2)	2.00–8.00	4.76 ± 1.65	0.333∗∗	**0.946**	–	–	–	–	–
Fertility knowledge (X3)	0.08–0.38	0.25 ± 0.08	0.393∗∗	0.374∗∗	**0.894**	–	–	–	–
Personality (X4)	0.00–0.47	0.21 ± 0.18	0.322∗∗	0.346∗∗	0.284∗∗	**0.954**	–	–	–
Perceived social support (Y1)	1.00–7.00	3.53 ± 1.11	0.263∗∗	0.196∗∗	0.242∗∗	0.272∗∗	**0.905**	–	–
E-Health literacy (Y2)	1.25–5.00	3.35 ± 0.95	0.361∗∗	0.339∗∗	0.413∗∗	0.317∗∗	0.345∗∗	**0.847**	–
Fertility motivation (Y3)	1.29–5.00	3.31–0.94	0.344∗∗	0.377∗∗	0.450∗∗	0.313∗∗	0.274∗∗	0.367∗∗	**0.904**

Diagonal bold numbers are AVE square root values, and numbers below the diagonal indicate Pearson’s correlation coefficients (∗∗∗*p* < 0.001∗∗). If the AVE square root value is greater than the correlation coefficients of the corresponding variable, it indicates good discriminant validity of the measured variable.

### Structural equation modeling

**Figure 2:**
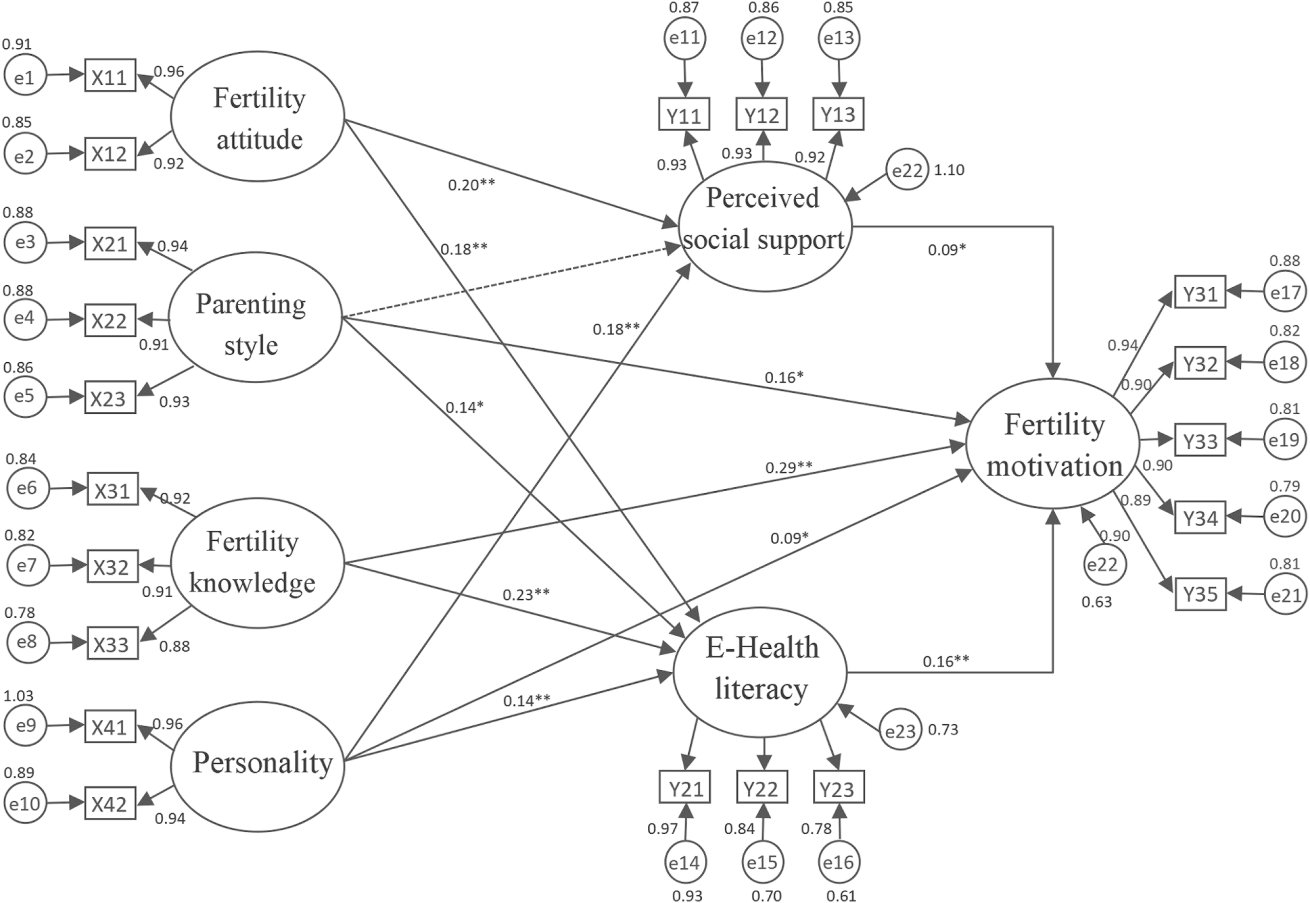
Structural equation model of college students' fertility motivation revised based on research samples and model parameters X11: positive; Y11: family support; X12: negative; Y12: friend support; X21: rejection; Y13: other support; X22: emotional warmth; Y21: application ability of test network health information and services; X23: over protection; Y22: judgment ability; X31: indicators of reduced fertility; Y23: decision-making ability; X32: misunderstandings about fertility; Y31: traditional concept category; X33: understanding of infertility; Y32: utility category; X41: introversion and extroversion; Y33: self-esteem category; X42: emotional stability; Y34: harmony category; Y35: love for children category.

Based on attribution theory, the theoretical framework of this study (Figure 1) includes 12 paths, with the analysis results shown in Figure 2. According to the modification indices suggested by AMOS 27.0, the hypothesized model was revised by removing the path from parenting style to perceived social support. After this adjustment, the final model comprised 11 paths. The fit indices for both the hypothesized and the revised models are presented in Table 5.

**Table 5:** Hypothesized model and modified model fit indices

	Item	Reference standards	Hypothesized model	Modified model
Absolute indexes	GFI	>0.900	0.906	0.915
	RMSEA	<0.080	0.047	0.048
Relative indexes	NFI	>0.900	0.907	0.937
	IFI	>0.900	0.912	0.930
	CFI	>0.900	0.912	0.920
	TLI	>0.900	0.899	0.915

The revised model demonstrated a better fit than the hypothesized model (Table 6). In this model, parenting style, fertility knowledge, personality, perceived social support, and e-Health literacy collectively explain 29.5% of the variance in fertility motivation. Perceived social support was explained by fertility attitude and personality, accounting for 9.5% of the variance. Online information was explained by fertility attitudes, fertility knowledge, and personality, accounting for 23.4% of the variance.

**Table 6:** Parameter estimates of variables for the modified model

		B (S.E.)	β	P	SMC
Fertility attitude	—E-Health literacy	0.196 (0.028)	0.195	<0.001	0.234
Fertility knowledge	—E-Health literacy	0.277 (0.028)	0.273	<0.001	–
Personality	—E-Health literacy	0.444 (0.063)	0.173	<0.001	–
Personality	—perceived social support	0.530 (0.075)	0.184	<0.001	0.095
Fertility attitude	—perceived social support	0.222 (0.031)	0.197	<0.001	–
Parenting style	—fertility motivation	0.171 (0.028)	0.158	<0.001	0.295
Personality	—fertility motivation	0.234 (0.061)	0.095	<0.001	–
Perceived social support	—fertility motivation	0.079 (0.02)	0.093	<0.001	–
E-Health literacy	—fertility motivation	0.153 (0.025)	0.159	<0.001	–
Fertility knowledge	—fertility motivation	0.286 (0.027)	0.294	<0.001	–

β, standardized estimate.

S.E., standard error; SMC, squared multiple correlation.

### Path analysis

In this study, the effects of the variables on fertility motivation showed differentiated patterns. Moreover, among the 11 paths included in the model, both direct and total effects were statistically significant (*p* < 0.05). The indirect effects of personality, fertility knowledge, and fertility attitude on fertility motivation were also statistically significant. Detailed results are presented in Table 7.

**Table 7:** Standardized direct, indirect, and total effects

Dependent variable	Independent variable	Direct effect	Indirect effect	Total effect
Fertility motivation	personality	0.095∗	0.040∗∗	0.135∗
	parenting style	0.178∗∗	–	0.178∗∗
	fertility knowledge	0.294∗∗	0.037∗∗	0.331∗∗
	fertility attitude	–	0.046∗∗	0.046∗∗
	e-health literacy	0.159∗	–	0.159∗
	perceived social support	0.093∗∗	–	0.093∗∗
Perceived social support	personality	0.184∗∗	–	0.184∗∗
	fertility attitude	0.196∗∗	–	0.196∗∗
E-Health literacy	personality	0.142∗∗	–	0.142∗∗
	parenting style	0.138∗	–	0.138∗
	fertility knowledge	0.231∗∗	–	0.231∗∗
	fertility attitude	0.177∗∗	–	0.177∗∗

∗*p* < 0.05, ∗∗*p* < 0.01, and ∗∗∗*p* < 0.001.

## Discussion

### Current status of college students’ fertility motivation

This study examined the factors influencing college students' fertility motivation. The analysis of sample characteristics shows that 33.2% of students explicitly chose “not to have children in the future,” which is consistent with the findings of Zhang et al.^33^ The scholar believes that this result reflects the “de-traditionalization” trend in contemporary fertility views among college students. Notably, more than half (51.5%) of the students are “uncertain about whether to have children in the future.” It can be seen that this group mostly exhibits a “wait-and-see” attitude in their fertility choices, and this phenomenon is more likely a comprehensive reflection of individuals' multiple factors, such as social support, fertility attitudes, and reproductive knowledge. The research results suggest that improving college students' cognitive literacy, guiding them to establish an objective and rational attitude toward childbearing, and enhancing their ability to perceive social support may effectively improve their level of fertility motivation.

Among the dimensions of fertility motivation, the “utility” dimension had the highest mean score. This result reflects that, as a group, college students may pay more attention to the practical effects of childbearing on aspects such as personal development and quality of life, such as the emotional value children provide and their role in fostering personal growth -rather than on the traditional expectation of continuing the family line. This result reflects that college students, as a group, may pay more attention to the practical effects of childbearing on personal development, quality of life, and other aspects.^34^ This finding is consistent with the existing research perspective on the value of children. Existing research on fertility values follows two paths; one is based on the value of children, which includes emotional value, economic value, and other aspects.^35^ In terms of emotional value, some scholars have found through research that children can also promote the harmonious development of marital relations,^35^ which also reflects that children have a certain emotional value in the intimate relationship between parents. Recent studies have shown that the depression level of the elderly with children in rural China is significantly lower,^36^ which further reflects that children also have a certain degree of spiritual support and emotional value for the elderly.^37^ From the perspective of the economic value of childbearing, scholars have found that the total expenditure of young families with two children is at least 322,060 yuan,^38^ and the increase in childbearing costs has gradually reduced its economic value. Overall, fertility motivation among college students was at a moderate level (79.45 ± 22.60 points), this is consistent with the findings of Duan,^39^ suggesting that there is a considerable potential fertility group. The results indicate that the fertility motivation level among college students still has room for improvement, and the future fertility population size is considerable.

### The fit of the structural model of college students’ fertility motivation

This study, grounded in Heider’s attribution theory, constructs a structural equation model. After modification, the model showed good fit indices (GFI = 0.915, RMSEA = 0.048, and so forth), confirming the theoretical adaptability. In this model, personality, parenting style, fertility knowledge, e-Health literacy, and perceived social support directly influenced fertility motivation (explaining 29.5% of the variance), suggesting that fertility motivation results from the combined effect of internal traits (such as personality and knowledge) and external environments (such as parenting style, e-Health literacy, and social support). Meanwhile, personality, fertility knowledge, and fertility attitude indirectly affect fertility motivation through “perceived social support” and “e-Health literacy.”

### Structural equation model of college students’ fertility motivation: Relationships among variables

This study revealed the complex interaction paths among variables and interpreted them in light of Heider’s attribution theory.

(1) The direct path of personality’s influence: Personality can directly affect fertility motivation; for example, an extroverted personality is more likely to accept the social value of childbearing. Experimental evidence from Norway indicates that extroverted men have a stronger fertility motivation than introverted men.^40^ In addition, not only does an individual’s own personality affect childbearing, but the partner’s personality also exerts a certain influence.^41^ On the other hand, personality can also function through an external attribution path: positive personalities such as high initiative promote “perceived social support,”^28^ which may enable individuals to more easily obtain positive feedback and support for childbearing from family and friends. Anxious traits such as neuroticism drive individuals to search for negative childbearing information from the Internet,^19^^,^^29^ ultimately exerting a reverse effect on fertility motivation.
(2) The pathway of fertility knowledge’s effects: Among the included variables, the standardized total correlation with the outcome variable fertility motivation is the greatest, its mechanism of action is divided into direct path and indirect path. In the direct path, reproductive knowledge can directly drive reproductive motivation. This conclusion is consistent with the research findings of foreign scholars. They discovered that the more reproductive knowledge one possesses, the stronger one’s reproductive motivation is.^26^ This may be because comprehensive reproductive knowledge may promote individuals to rationally evaluate the feasibility of childbearing. The results of a randomized controlled trial showed that the group receiving reproductive knowledge education intervention achieved fertility faster two years later than the group not receiving the intervention.^42^ Another study on American students pointed out that this group has limited mastery of fertility-related knowledge but a strong learning tendency, which also indicates that it is feasible to carry out fertility education intervention at the undergraduate stage.^43^ Meanwhile, it can also exert an indirect effect through e Health literacy.
(3) Indirect transmission pathway of fertility attitude: Fertility attitude did not directly determine fertility motivation, but exerted indirect effects through perceived social support and e-Health literacy. This result may be due to the fact that individuals with a positive attitude toward procreation are more likely to perceive the support of their partners, elders, or friends when making decisions related to reproduction. Moreover, they also understand that social support has a positive impact on reproductive motivation,^23^ this positive perception of social support then transforms into a positive force that drives the motivation for having children. On the other hand, fertility attitudes influenced the type of electronic health resources individuals accessed,^22^ and may consequently exert a certain indirect action on fertility motivation through the processes of information perception and cognitive processing.
(4) In this study, the path from parenting style to perceived social support was not significant. In this study, the main reason for the cognitive changes during college students' adulthood is their growing maturity and independence. As they grow older and become more independent, college students' perception of social support shifts from past dependence on their families to multiple channels such as classmates, friends, and campus resources. During this transformation process, parental support still holds a certain position, but the influence of the “parental parenting style” may have been diluted by various factors.

In summary, “perceived social support” and “e-Health literacy” emerged as core mediating variables. It is recommended that the government strengthen the perception of social support by promoting fertility policies such as expanding community childcare services. Universities should integrate network literacy education into their curricula, offering courses, such as “scientific fertility”—to guide students in rationally evaluating health information. Ultimately increasing the likelihood of multi-channel ultra-high fertility motivation.

### Practical insights

Based on the above, this study focuses on cultivating college students’ fertility motivations and explores potential directions for future implementation from three dimensions: individual, university, and policy. The following practical directions are exploratory in nature, and their actual implementation effects remain to be tested and validated through practice and research.

(1) Individual students can enhance their knowledge reserve on fertility and e-Health literacy. As the primary decision-makers for future childbearing, university students should acquire scientific knowledge on fertility, including topics such as physiological health and prenatal/postnatal care. Simultaneously, given the complexity of e-Health resources, it is also essential to improve e-Health literacy by learning to critically evaluate, discern, and utilize electronic resources to avoid being misled by one-sided or negative information.
(2) Higher education departments should innovate fertility education models and implement differentiated interventions for college students with varying characteristics. Universities may offer public courses on fertility education, integrating modules such as fertility knowledge dissemination, policy interpretation, and workplace reproductive rights analysis. They could also leverage digital resource platforms such as short videos and online forums, or organize diverse activities such as parenting simulation experiences to enhance the engagement and appeal of fertility education. For students with distinct personality traits or family upbringing backgrounds, tailored approaches to activate fertility motivation should be developed. For instance, students exhibiting pronounced neurotic traits may require focused interventions strengthening social support systems, while those from adverse upbringing environments may benefit from additional emotional support and psychological counseling.
(3) In terms of policy precision, the government and society can pilot academic-fertility linkage policies targeting college students, such as establishing childcare grants for university students and implementing flexible childcare systems in internship organizations, thereby reducing students' perceived conflict between childbearing and individual Homo sapiens development. Simultaneously, collaborative efforts with enterprises to launch a “College Student Fertility-Friendly Internship Program” could be introduced, for instance, by adding parental leave during internships to proactively foster a fertility-friendly social environment.

### Limitations of this study

(1) Sample representativeness Bias: There is a certain gender imbalance in the sample, which may be related to factors such as the survey channels and the willingness of the respondents to participate. This limitation may, to some extent, affect the extrapolation scope of the research conclusions. Future studies can adopt more rigorous stratified sampling, optimize the gender structure of the sample, and enhance the external validity of the conclusions.
(2) Boundary of causal inference: This study is a cross-sectional study. The research results can only reveal the correlation between variables and cannot determine a strict causal direction. If more comprehensive influencing factors are included in the future, the relationship between the research results and the relative importance of variables may change. We have adjusted the conclusion statement accordingly and strictly defined the scope of the research inference. The cross-sectional correlation findings of this study provide clear hypotheses and variable foundations for subsequent verification of causal directions through cross-lagged models, instrumental variables, or quasi-experimental designs.
(3) Lack of dynamism: As a cross-sectional survey, this study only captured individuals’ static fertility motivation and was unable to track changes caused by key life choices such as employment, romantic relationships, and marriage. Future research should use longitudinal surveys to systematically collect data on factors such as work intensity, career development, partner attitudes, and marital status, thereby revealing the developmental trajectory of fertility motivation and improving the explanatory power of the model.

## Resource availability

### Lead contact

Requests for further information and resources should be directed to and will be fulfilled by the lead contact, Yongai Zhang (zhangyongai@xiyi.edu.cn).

### Materials availability

This study did not generate new unique reagents.

### Data and code availability

• Data reported in this paper will be shared by the lead contact upon request.
• This paper does not report original code.
• Any additional information required to reanalyze the data reported in this paper are available from the lead contact upon request.

## Acknowledgments

This study was supported by the “Support Plan for Sanqin Scholars Innovation Team,” Shaanxi Province ([2020]45). Another is “A Multidimensional Empirical Study on Psychological Health Factors in Patients with Recurrent Miscarriage: An SEM-Based Approach” (2025HXSK10).

## Author contributions

Conceptualization, Z.G.; methodology, Z.G. and Y.Z.; investigation, Z.G., Y.Z., and X.Z.; writing – original draft, Z.G., Y.Z., and X.Z.; writing – review and editing, Z.G., Y.Z., X.Z., and W.L.; funding acquisition, Y.Z.; supervision, Z.G., Y.Z., X.Z.,W.L., and L,.S.

## Declaration of interests

The authors declare no competing interests.

## STAR★Methods

### Key resources table

**Table:** 

REAGENT or RESOURCE	SOURCE	IDENTIFIER
**Software and algorithms**		

IBM SPSS Statistics 26	IBM, Armonk, N.Y., USA	https://www.ibm.com/cn-zh/spss
IBM SPSS AMOS 27	IBM, Armonk, N.Y., USA	https://www.ibm.com/cn-zh/products

### Method details

#### Study design

This study adopted a cross-sectional survey design. Based on the regional classification of China into four major sections -eastern, central, western, and northeastern - 12 universities representing five distinct regions (north China, east China, northwest China, northeast China, and central China) were selected as the survey sites. A structural equation model (SEM) was used to analyze the direct and indirect interaction pathways among fertility attitude, parenting style, fertility knowledge, personality, perceived social support, eHealth literacy, and fertility motivation, with the aim of clarifying the intrinsic mechanisms underlying these variables.

#### Sample and data collection

This study was conducted from July to August of 2025, using a multi-stage stratified cluster sampling method.

(1) Geographical Stratification: Based on the National Bureau of Statistics’ division of mainland China into four major economic regions (eastern, central, western, and northeastern), and considering regional social, cultural, and demographic characteristics, nine provinces, municipalities, or autonomous prefectures were selected as sampling units. Specifically, these included: the eastern region (Shandong, Hebei, and Beijing), the central region (Henan, Jiangxi), the western region (Shaanxi, Shanxi), and the northeastern region (Jilin, Yanbian Korean Autonomous Region). Yanbian was separately included due to its distinctive ethnic population characteristics, ensuring the cultural represent ativeness of the sample.
(2) Institution Sampling: From the mentioned regions, 12 comprehensive universities were randomly selected, covering various academic disciplines such as humanities, science and engineering, social sciences, and medicine, to minimize systematic bias related to academic background.
(3) Subject Selection: Within each selected universities, “classes” served as cluster units. At least four full-time undergraduate classes were randomly chosenfrom each university, and all students in the selected classes who met the inclusion criteria were invited to participate.

The research team contacted university officials to explain the purpose, significance, and data collection process. Once they agreed, the research team trained them as investigators under a standardized research protocol.These university-based investigators then briefed the students on the study and the survey requirements. After informed consent was secured from the participants, the electronic questionnaires were distributed via the “Questionnaire Star” platform and configured to allow only one submission per IP address. All questions were set as mandatory to prevent missing data. This research questionnaire comprises a total of 142 items, and the estimated completion time is 10 min. Prior to the survey, the participants were clearly informed of the questionnaire’s duration and the research process. A total of 2,010 questionnaires were distributed, and 1,550 valid questionnaires were retrieved, with an effective recovery rate of 77.1%.

The specific inclusion and exclusion criteria for this study are as follows.

(1) Inclusion criteria: Participants must be 18 years of age or older, currently enrolled as a full-time undergraduate student, informed about the study, and willing to participate.
(2) Exclusion criteria: Inability to understand or complete the survey independently, communication difficulties,or poor compliance.

#### Instruments

##### General information questionnaire

This study was conducted through literature research and expert consultation. The general information questionnaire was independently designed by the researchers and the research team.It mainly covered demographic and sociological data, including gender, age, whether one is an only child, household registration type, total family size, academic major classification, and current attitude toward childbirth.

##### Fertility attitude scale

This study utilized the well-established scale developed by Billar.^44^ to measure fertility attitudes across two dimensions (positive and negative) with a total of 12 items. The original scale’s Cronbach’s α coefficient was 0.853.The original foreign English scale used in this study has obtained translation authorization. The research team completed the Chinese adaptation following the standard translation-back-translation procedure. After independent translation by bilingual researchers, back - translation by native experts, semantic comparison, and cultural adaptation, the official Chinese version was formed. It adopts the Likert 5 - point scoring system, with 1–5 representing: “Strongly disagree”, “Somewhat disagree”, “General”, “Somewhat agree”, “Completely agree”. This study conducted an internal consistency reliability test on the Chinese version of the scale, with a Cronbach’s α coefficient of 0.969, indicating good reliability of the scale and its suitability for this study.

##### Chinese Short-Form Egna Minnen av Barndoms Uppfostran (EMBU) parenting style questionnaire

This study employed the revised Chinese version of the Short-Form EMBU parenting style scale by Jiang.^45^ The scale has three dimensions: emotional warmth, over protection, and rejection, with a total of 21 items. This scale employs the Likert 4-point scoring system, with scores ranging from 1 to 4, representing “never”, “occasionally”, “often”, and “always” respectively. The total scores of the participants on each dimension are calculated. The original scale’s Cronbach’s α coefficient was 0.840; in this study, it was 0.900.

##### Chinese version of the Cardiff Fertility Knowledge Scale

This study used the Chinese-translated Cardiff Fertility Knowledge Scale (CFKS) by Zhou^46^ to assess fertility knowledge among individuals of reproductive age. The scale covers three dimensions: indicators of reduced fertility, misconceptions about fertility, and understanding of infertility, with a total of 13 items. This scale has standard answers. There are 8 positive-score items and 5 negative-score items. It uses 3 evaluation options: “correct”, “wrong”, and “don’t know”. A score of 1 is given for correct answers, 0 for incorrect answers (including “don’t know”), and the total score as well as the percentage of the total score represent the score of this scale. The score ranges from 0% to 100%. The higher the score, the better one’s mastery of reproductive-related knowledge. The original scale’s Cronbach’s α coefficient was 0.811; in this study, it was 0.960.

##### Eysenck Personality Questionnaire short scale

This study utilized the Chinese version of the Eysenck Personality Questionnaire (EPQ) revised by Chen^47^ and others, specifically the Extraversion scale (E scale) and the Neuroticism scale (N scale), comprising a total of 45 items. This scale is a dichotomous scale. A score of 1 is given for “yes” and 0 for “no”. The total score for each dimension is obtained by simply adding up the scores of the corresponding items. A high score on the E scale indicates a higher level of extraversion, while a high score on the N scale indicates a weaker emotional stability. Theorginal scale’s Cronbach’s α coefficient is 0.88; in this study, it was 0.910.

##### Perceived Social Support Scale

This study employed the Perceived Social Support Scale revised by Jiang.^48^ The scale covers three dimensions: family support, friend support, and other support, with a total of 12 items. This scale adopts the Likert 7-point scoring system, with 1–7 representing “extremely disagree”, “very disagree”, “slightly disagree”, “neutral”, “slightly agree”, “very agree”, and “extremely agree” respectively. The score range of the scale is 12–84 points. The higher the score, the greater the degree of subjective social support that the individual feels. The original scale’s Cronbach’s α coefficient scale is 0.899; in this study, it was 0.970.

##### e-health literacy scale

It refers to the comprehensive ability of an individual to search for, obtain, understand, evaluate health information through electronic channels, and process and apply this information to solve health-related problems.

This scale was developed and compiled by Norman^49^ and others, measuring three dimensions: the ability to use online health information and services, judgment, and decision-making, with a total of 8 items. In this study, the Chinese version of the electronic health literacy scale, which was translated and adapted by Chinese scholar Guo Shuaijun,^50^ was used. The Likert 5-point scoring system was employed, where 1 to 5 represent “very inconsistent”, “somewhat inconsistent”, “unclear”, “somewhat consistent”, and “very consistent” respectively. The higher the score, the higher the level of electronic health literacy. The Chinese version of this scale has a Cronbach’s α of 0.913; in this study, it was 0.936.

##### Fertility motivation scale

This study used the Fertility Motivation Questionnaire for Young People of Childbearing Age developed by Fang et al.^51^ in 2018. The questionnaire includes five dimensions: traditional beliefs, life harmony, love for children, self-esteem, and utility, with a total of 24 items. This scale uses a 5-point Likert scoring system, where 1 to 5 represent “strongly disagree”, “disagree”, “uncertain”, “basically conforms”, and “completely conforms” respectively. The higher the score, the stronger the motivation for having children. The original scale’s Cronbach’s α coefficient was 0.848, and in this study, it was 0.950.

##### Data analysis

Data were collected via the Questionnaire Star platform and directly exported from the platform. SPSS 26.0 was used for descriptive statistics and correlation analysis Cronbach’s α coefficient was used to evaluate the reliability of each scale. Continuous variables were expressed as mean ± standard deviation ($\bar{x}$ ± s), while categorical variables were described by frequency and proportion.

AMOS 27.0 was utilized to validate and refine both the measurement and structural models. Reliability was assessed through confirmatory factor analysis (CFA), and discriminant validity of the measurement model was tested with Pearson correlation coefficient. Model fit was evaluated using CFI, NFI, TLI, and RMSEA. Indirect, direct, and total effects, along with their significance, were estimated via bootstrapping. The Bootstrap ML method^52^ was applied in the study, and it can yield more stable results and is considered more accurate for sample sizes greater than 200.^53^ A 95%-corrected confidence interval was employed with 5,000 bootstrap resamples, and significance was set at *p* < 0.05.

##### Ethics

This study protocol has been approved by the Ethics Committee of Xi’an Medical University (Approval No.: XYLS2025136). Written informed consent was obtained from all participants.

### Quantification and statistical analysis

Statistical analyses were performed using IBM SPSS Statistics and AMOS.
